# Exploring Amantadine Derivatives as Urease Inhibitors: Molecular Docking and Structure–Activity Relationship (SAR) Studies

**DOI:** 10.3390/molecules26237150

**Published:** 2021-11-25

**Authors:** Atteeque Ahmed, Aamer Saeed, Omar M. Ali, Zeinhom M. El-Bahy, Pervaiz Ali Channar, Asma Khurshid, Arfa Tehzeeb, Zaman Ashraf, Hussain Raza, Anwar Ul-Hamid, Mubashir Hassan

**Affiliations:** 1 Department of Chemistry, Quaid-I-Azam University, Islamabad 45320, Pakistan; aahmed@chem.qau.edu.pk (A.A.); pervaizali@chem.qau.edu.pk (P.A.C.); asmakhurshid@chem.qau.edu.pk (A.K.); 2 Department of Chemistry, Turabah University College, Turabah Branch, Taif University, P.O. Box 11099, Taif 21944, Saudi Arabia; om.ali@tu.edu.sa; 3 Department of Chemistry, Faculty of Science, Al-Azhar University, Nasr City, Cairo 11884, Egypt; zeinelbahy@azhar.edu.eg; 4 Department of Pharmacy, Quaid-i-Azam University, Islamabad 45320, Pakistan; arfatehzeeb@gmail.com; 5 Department of Chemistry, Allama Iqbal Open University, Islamabad 44000, Pakistan; zaman.ashraf@aiou.edu.pk; 6 Department of Biological Sciences, College of Natural Sciences, Kongju National University, 56 Gongjudehak-Ro, Gongju 314-701, Chungnam, Korea; hussain_solangi@yahoo.com; 7 Core Research Facilities, King Fahd University of Petroleum and Minerals, Dhahran 31261, Saudi Arabia; anwar@kfupm.edu.sa; 8 Institute of Molecular Biology and Biotechnology, The University of Lahore, Lahore 54000, Pakistan; mubashirhassan_gcul@yahoo.com

**Keywords:** acyl/aroyl thioureas, urease inhibitors, synthesis, enzyme inhibitory kinetics, molecular docking

## Abstract

This article describes the design and synthesis of a series of novel amantadine-thiourea conjugates (**3a**–**j**) as Jack bean urease inhibitors. The synthesized hybrids were assayed for their in vitro urease inhibition. Accordingly, *N*-(adamantan-1-ylcarbamothioyl)octanamide (**3j**) possessing a 7-carbon alkyl chain showed excellent activity with IC_50_ value 0.0085 ± 0.0011 µM indicating that the long alkyl chain plays a vital role in enzyme inhibition. Whilst *N*-(adamantan-1-ylcarbamothioyl)-2-chlorobenzamide (**3g**) possessing a 2-chlorophenyl substitution was the next most efficient compound belonging to the aryl series with IC_50_ value of 0.0087 ± 0.001 µM. The kinetic mechanism analyzed by Lineweaver–Burk plots revealed the non-competitive mode of inhibition for compound **3j**. Moreover, in silico molecular docking against target protein (PDBID 4H9M) indicated that most of the synthesized compounds exhibit good binding affinity with protein. The compound **3j** forms two hydrogen bonds with amino acid residue VAL391 having a binding distance of 1.858 Å and 2.240 Å. The interaction of **3j** with amino acid residue located outside the catalytic site showed its non-competitive mode of inhibition. Based upon these results, it is anticipated that compound **3j** may serve as a lead structure for the design of more potent urease inhibitors.

## Highlights:

* Novel amantadine-thiourea molecular hybrids (**3a**–**j**) synthesized and assayed for in vitro urease inhibition.
* Compound **3j** with 7-carbon alkyl chain showed excellent activity (IC_50_ 0.0085 ± 0.0011 µM), and **3g** bearing a 2-chlorophenyl substitution was the next most effective (IC_50_ 0.0087 ± 0.0011 µM)
* Molecular docking: molecular dynamic simulations and kinetic studies support **3j** as the lead structure for the design of more potent inhibitors

## 1. Introduction

Urease or urea amidohydrolase is a metalloenzyme that holds two Ni^+2^ ions in its active site and is found in a variety of bacteria, fungi, algae, plants, certain invertebrates, soil, and ruminants. The high rate of urea hydrolysis in the rumen makes efficient ammonia assimilation in order to challenge microbial protein. Urease inhibitors limit the action of urease, allowing microorganisms and mammals to better utilize urea. As the amount of NH_3_ increases, the pH rises, allowing *Helicobacter pylori*, the bacteria that causes peptic ulcers, ulcerative colitis, and stomach cancer, to survive. [1,2]

Urease, is a nickel-dependent metalloenzyme found in plants, bacteria, and fungi. Bacterial enzyme is particularly important because it has been shown to be a powerful virulence factor in various species. It is particularly important for *Helicobacter pylori* metabolism and virulence, as both are required for penetration of the stomach mucosa, and it is a potent immunogen that evokes an effective immune response. As a result, it is no surprise that attempts to design, produce, and analyze novel urease inhibitors are a hot topic in biomedical sciences.

Regardless of the implications of urease inhibitors, only one compound, acetohydroxamic acid, has been approved for human or animal usage, but its use is limited due to its negative side effects, which include kidney stones, deep-vein phlebothrombosis, and lower-extremity phlebitis [3,4]. Thiourea and its functionalized derivatives are the most studied urease inhibitors due to their structural similarity to urease substrate. Ni^+2^ ions are known to be chelated by *N*-substituted thioureas, and a number of substituted *N*-aryl acetothiourea derivatives have antibacterial and urease inhibitory activities [5,6].

Amantadine (1-aminoadamantane) is a commonly used medicine for the treatment of Parkinson’s disease-related dyskinesia and influenza caused by influenza virus type A. It works as a nicotinic antagonist as well as a noncompetitive *N*-Methyl-D-aspartate (NMDA) antagonist, making it useful in the treatment of Alzheimer’s disease [7]. Vaccinia virus is inhibited by different amino acetyl adamantyl amines [8], and 1-adamantane carboxylic acid amides are potent inhibitors of smallpox virus and, more recently, against several coronaviruses [9].

**Figure 1:**
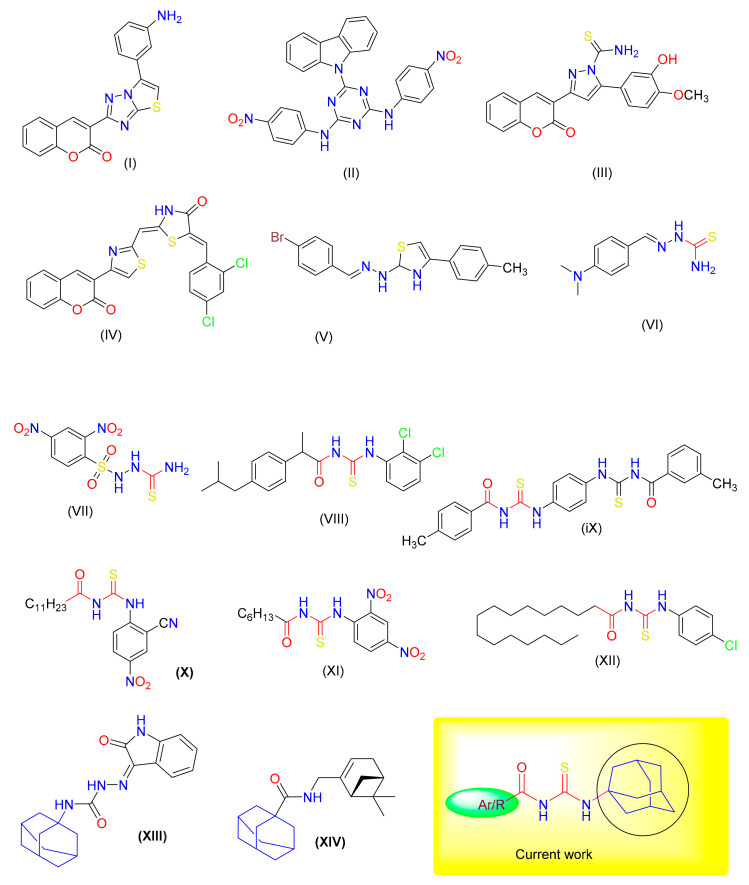
Previously reported inhibitors of Jack Bean Urease (I–XII); some anti-viral adamantane derivatives (XIII–XIV) and design of current work.

Previously, we have reported several small molecules possessing significant urease inhibitory activities It is evident that a majority of them are thioureas-based molecules Figure 1 [10,11,12,13,14,15,16,17]. The presence of adamantyl moiety further improves efficacy; therefore, in the present article, preparations of new amantadine-thiourea hybrids and inhibitory effects of various functionalities on urease activity have been reported [18].

The important structural features of our designed molecules include the presence of an alkyl and aryl substituent for structural variation, thiourea linkage which resembles the substrate of urease enzyme, and appearance of an adamantyl group. The significance of compounds bearing an adamantyl substituent in drug design has been well documented. These compounds have demonstrated their efficacy in the treatment of neurological conditions as anti-viral agents and as anti-type 2 diabetic agents. The large hydrophobic constant for the adamantyl group (n = 31) can shift the high-water solubility of a compound to the clinically desirable range. The steric bulk of the adamantyl group can either restrict or moderate intramolecular reactivity or can hinder the access of hydrolytic enzymes, thus increasing drug stability and plasma half-life [19].

## 2. Results and Discussion

**Figure 2:**
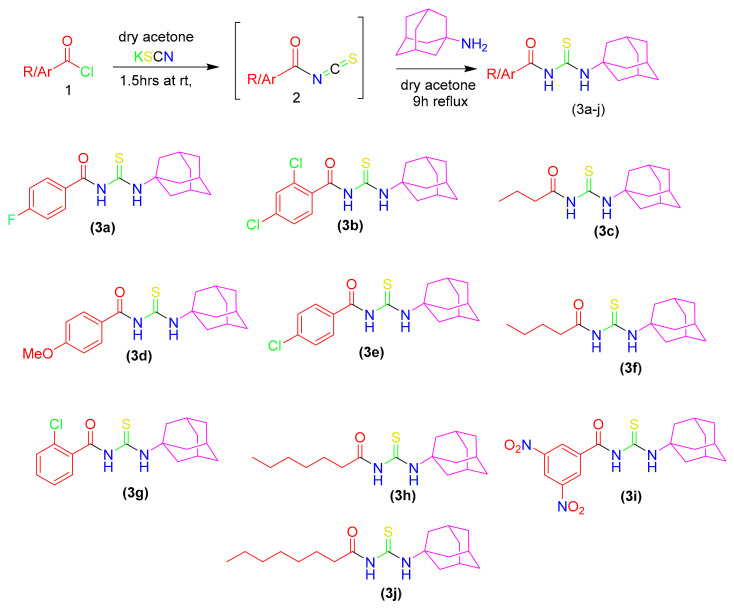
Synthetic scheme and molecular structures of synthesized derivatives.

Figure 2 depicts the synthetic pathway used to synthesize the title compounds. Aroyl isothiocyanates (**1a**–**j**) were reacted in situ with an equimolar ratio of amantadine in dry acetone in order to provide the required amantadine-thiourea conjugates (**3a**–**j**) in high yields and purity.

The structure of all newly synthesized thiourea derivatives was established via spectroscopic analysis. In ^1^H NMR, two distinctive signals for N-H protons were observed at δ 11.50 and 10.67 ppm for compound **3b.** The more de-shielded signal at δ 11.50 was attributed to N-H proton for nitrogen atom fringed by both carbonyl and thiocarbonyl carbons. The N-H proton appeared at δ 10.67 ppm because nitrogen is further attached to thiocarbonyl carbon. The difference in chemical shift values was due to the electrophilic effect of carbonyl and thiocarbonyl functional groups. The aromatic protons in the structures appeared in their characteristic regions around δ 7.72–7.52 ppm. The signals ranging from δ 2.26 to 1.66 ppm were due to the protons attached to sp^3^ hybridized carbons of the adamantyl group. In ^13^C NMR, distinctive signals appeared at δ 177.65 and δ 167.57 for thiocarbonyl and carbonyl carbons, respectively. The signals at δ 136.16–127.7 ppm were assigned to aromatic carbons whilst the signals for sp^3^ hybridized carbons of the adamantyl group were assigned in their characteristic region. Tertiary carbon directly attached to nitrogen appeared at δ 54.90 ppm while that at δ 40.11 assigned to -CH_2_-carbons next to the tertiary carbon. The signal at δ 36.20 ppm was for -CH-carbons next to the tertiary carbon whilst signal at δ 29.29 ppm for -CH_2_- of adamantyl group.

### 2.1. In Vitro Urease Inhibitory Activity and Structure Activity Relationship (SAR)

Table 1 shows in vitro urease inhibitory activity of the synthesized compounds (**3a**–**j**). According to the SAR data, all of the newly synthesized compounds were more potent than the conventional standard inhibitor thiourea. The adamantyl derivatives of alkyl chains were found to be more active than their aryl analogues. The aryl molecule **3d**, which has a 2-chloro group, was found to be the most efficient inhibitor of urease, followed by **3a**, which has a flouro group at the four position. In aliphatic chain bearing compounds, it was noted that there was a small difference in efficacy in proceeding from propyl to butyl, whilst it increased considerably in the case of hexyl to heptyl chains, and the latter is the most active in the series. This indicates that the length of alkyl chain influences the activity.

### 2.2. Kinetic Analysis

**Figure 3:**
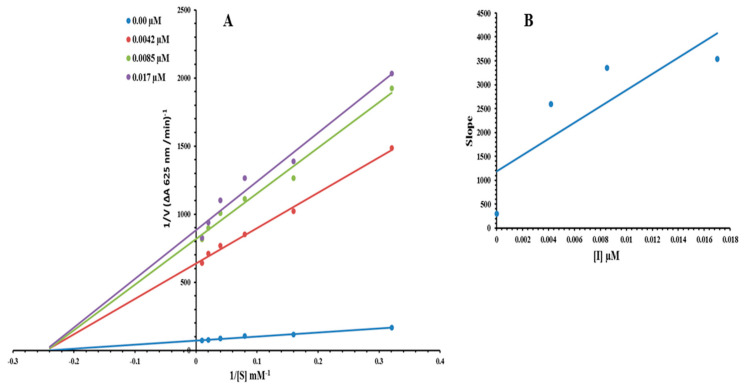
Lineweaver-Burk plots for inhibition of urease in the presence of compound **3j**. (**A**) Concentrations of **3j** were 0.00, 0.0042, 0.0085, and 0.017 µM. (**B**) The insets represent the plot of the slope or the vertical versus inhibitor **3j** concentrations to determine inhibition constants. The lines were drawn using linear least squares fit.

Based on our results, we selected the most potent compound **3j** for the determination of inhibition type and inhibition constant. The kinetic studies of the enzyme by the Lineweaver–Burk plot of 1/V versus 1/[S] in the presence of different compound concentrations produced a series of straight lines (Figure 3A). The results of compound **3a** showed that compound intersected within the second quadrant. The analysis showed that Vmax decreased with increasing inhibitors concentration whilst Km remains constant. Thus, compounds **3f** inhibit the urease non-competitively in order to form an enzyme inhibitor complex. A secondary plot of the slope against the concentration of inhibitors showed an enzyme inhibitor dissociation constant (K*i*) (Figure 3B). The kinetic results are presented in the Table 2.

### 2.3. Free Radical Scavenging

The synthesized thiourea derivatives were evaluated for DPPH free radical scavenging potential. All of the synthesized compounds (**3a**–**j**) exhibited smaller radical scavenging activity compared to the standard (ascorbic acid), even at the high concentrations (100µg/mL) Table 3.

### 2.4. Structural and Physiochemical Evaluation of Jack Bean Urease

**Figure 4:**
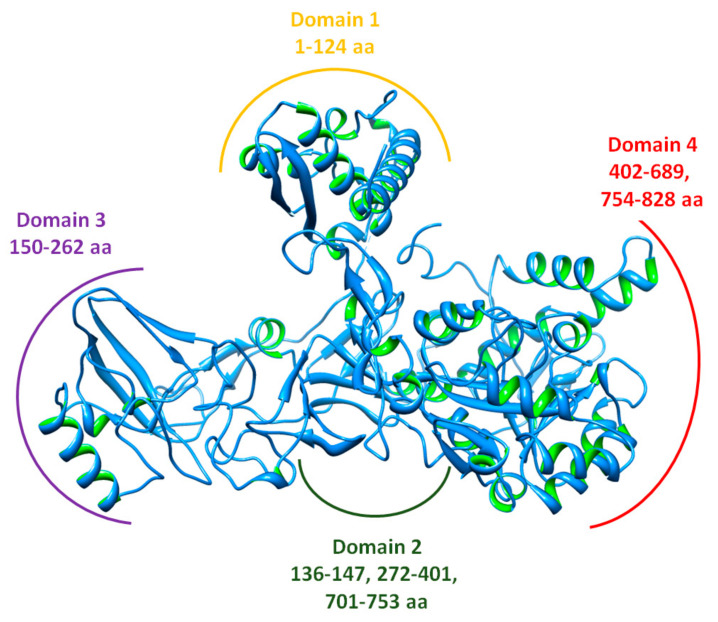
Protein structure with domain residues.

The overall protein structure of Jack bean urease is mentioned in Figure 4. The VADAR analysis showed protein architecture possessing 27% helices, 31% β sheets, and 41% coils in the target protein. Ramachandran plots suggested that 97.5% of residues were present in favored regions, which shows the precision of phi (φ) and psi (ψ) angles among the coordinates of jack bean urease. The physiochemical properties (Table 3) of jack bean urease such as molecular weight (Mw) were calculated by aggregation of average isotopic masses of residues. On the other hand, the theoretical pI was computed by using pK values of amino acids of the target protein [20]. A literature study revealed the computational predicted pI range values (4.31 to 11.78) of proteins [21]. The predicted pI value 6.05 is comparable with the standard values, indicating the accuracy of targeted protein. The GRAVY value is the sum of hydropathic values of all amino acids present in the protein [22], although increasing negative values indicate that it is more hydrophilic and less hydrophobic in behavior.

### 2.5. Molecular Docking Analysis

#### 2.5.1. Binding Energy Evaluation of Synthesized Compounds

In order to predict the best-fit conformational position of synthesized compounds (**3a**–**j**), these were docked against urease. The generated docked complexes were examined based on glide docking energy values (Kcal/mol) and bonding interaction (hydrogen/hydrophobic) pattern. The binding energy value depicts the best conformational position within the active region of target protein. The docking results indicate that ligand **3i** revealed the best energy value (−5.122 Kcal/mol) compared to all other ligands Table 4. The in vitro and in silico results showed a good correlation and in both experiment data.

#### 2.5.2. Binding Pocket Analysis

**Figure 5:**
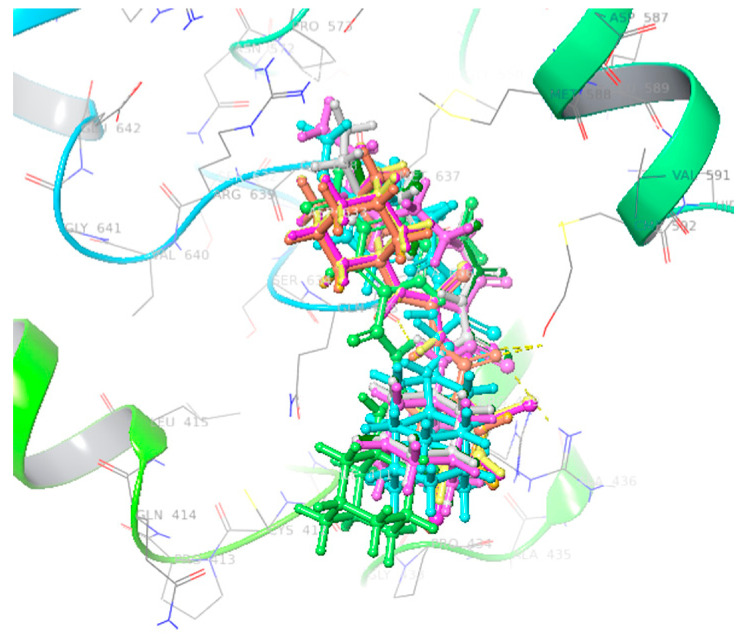
Docking complexes of all ligands within active region having similar conformations.

Docking analysis indicated that all compounds (**3a**–**j**) demonstrated binding interactions in the binding pocket of target protein with comparable conformational poses. Figure 5 shows that all ligands are actively confined within the binding pocket and involved in interactions with amino acid residues, predicting their significance against urease.

#### 2.5.3. Ligand-Binding Analysis of Urease Docked Complexes

**Figure 6:**
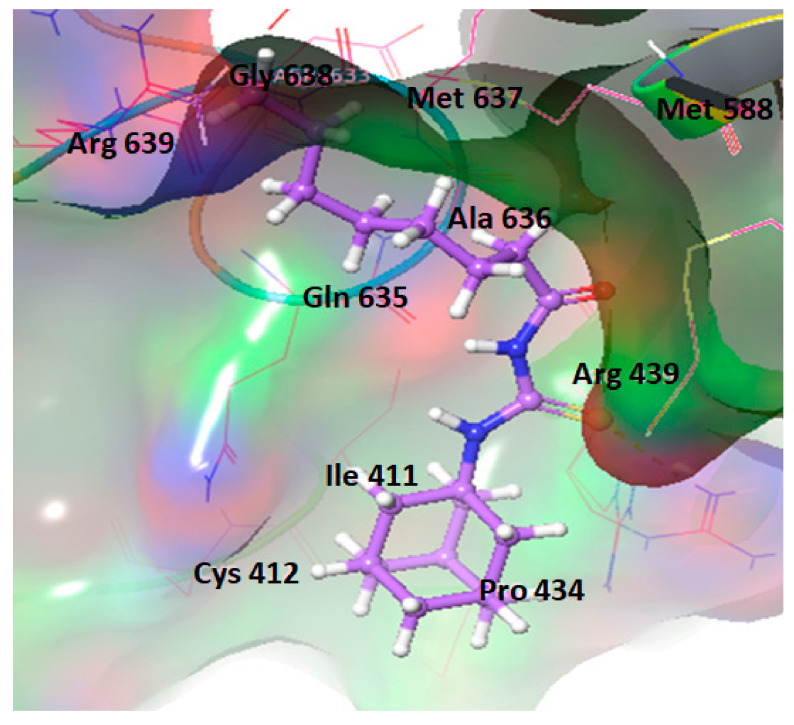
Docking complex of **3j**.

**Figure 7:**
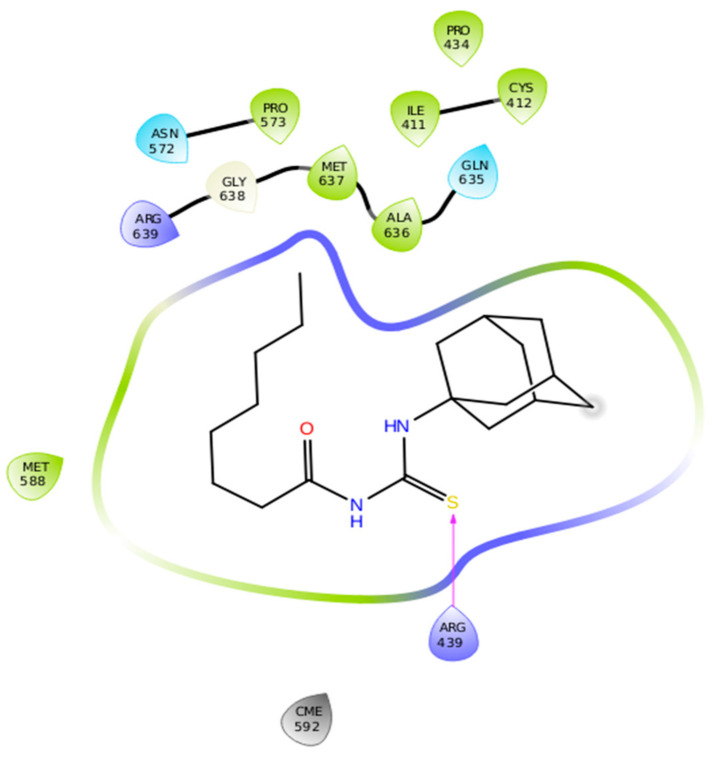
2D depiction of **3j** docking complex.

A thorough SAR study showed that single active H-bonds were observed in **3j** against the target protein in docking complex. The thiourea derivative showed H-bond with amino acid residue Arg439. The other residues such as Met588, Arg638, Met637, Ala636, and Gln635 are also part of binding pocket residues and close to **3j** hydrophobic interactions (Figure 6). The 2D depiction of **3j** docking complex is mentioned in Figure 7**.** Literature data also confirmed the importance of these residues in bonding with other urease inhibitors [23,24,25,26,27]. Comparative binding energy and SAR analysis showed that compound **3j** may be thought as potent inhibitor of jack bean urease. The other docking complexes are provided in the Supplementary Materials.

**Figure 8:**
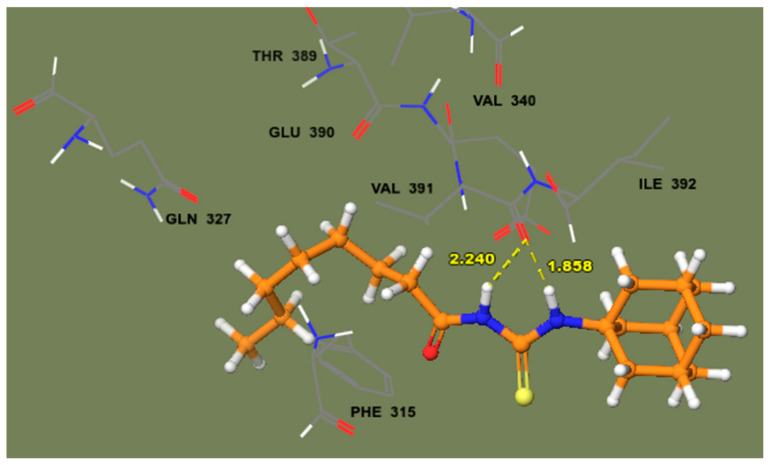
The docking complex of **3j** with urease enzyme (PDBID 4H9M) showed binding interaction with VAL391 located outside the catalytic site of enzyme.

The docking of the most potent derivative **3j** has been performed against target enzyme PDBID 4H9M in order to find a binding pocket other than the catalytic site to assure a non-competitive binding mode with respect to the inhibitor. It has been observed that -NH of thiourea moiety of the compound **3j** forms two hydrogen bonds with amino acid residue VAL391 having binding distance 1.858 Å and 2.240 Å (Figure 8). As the most potent derivative **3j** binds well with the amino acid located outside the enzymatic catalytic pocket, we may, thus, propose that it is non-competitive mode of inhibition.

## 3. Experimental

### 3.1. Materials and Methods

All reagents were purchased from Sigma Aldrich and used without further purification. Melting points were determined on a Stuart SMP3 melting point apparatus. The NMR spectra were recorded on a Bruker 300 (Billerica, Massachusetts, United States) (^1^H-NMR at 300 MHz and for ^13^C-NMR at 75.5 MHz), and chemical shifts are reported in parts per million (ppm) versus tetramethyl silane or the residual solvent resonance as an internal reference standard. The reactions were monitored by using thin layer chromatography (TLC), and it was performed using aluminum sheets coated with silica gel F254 (Merck, Darmstadt, Germany) detection using UV light at 254 and 360 nm.

### 3.2. General Procedure

With respect to the stirred solution of potassium thiocyanate (5.5 mmol) in dry acetone (15 mL), freshly prepared substituted benzoyl chlorides (5.0 mmol) (**1a**–**j**) were added dropwise under an inert environment for 1.5 h. The formation of acyl thiocyanate intermediates was shown by the appearance of a milky color of solution. The reaction mixture was cooled at room temperature, and a solution of amantadine (**2**) (5.0 mmol) in dry acetone was added dropwise, and the reaction mixture stirred further for 5–6 h at room temperature. Upon completion, the reaction mixture was added to crushed ice. The solids precipitated were filtered, washed with cold water, dried, and recrystallized from ethanol to furnish the target molecules (**3a**–**j**).

*N*-(Adamantan-1-ylcarbamothioyl)-4-fluorobenzamide (**3a**):

White crystalline solid, m.p. = 180–182 °C, Yield = 86%, R_f_ = 0.71 (n-Hexane: Ethyl acetate 4:1) ^1^H NMR (DMSO-d_6_, 300 MHz,); δ (ppm): 11.01 (s, 2H, NH), 7.98 (d, 2H, *J* = 8.7 Hz Ar-H), 7.32 (d, 2H, *J* = 8.7 Hz, Ar-H), 2,27 (m, 6H, CH_2_), 2.09 (m, 3H, CH), 1.66 (m, 6H, CH_2_); ^13^C NMR (75 MHz DMSO-d_6_) δ (ppm) 178.01 (C=S), 167.88 (C=O), 132, 131.88, 129.26, 129.22, 116, 115.70 (Ar-C), 54.75, 40.17, 36.25, 29.2 (adamantyl-C) Anal. calcd. for C_18_H_21_FN_2_OS (332.14): C, 65.03; H, 6.37: F,5.71 N, 8.43: S, 9.64. Found: C, 65.04; H, 6.38; N, 8.44.

*N*-(Adamantan-1-ylcarbamothioyl)-2,4-dichlorobenzamide (**3b**):

White crystalline solid, m.p. = 204–205 °C, Yield = 81%, R_f_ = 0.56 (n-Hexane: Ethyl acetate 4:1) ^1^H NMR (DMSO-d_6_, 300 MHz,); δ (ppm): 11.50 (s, 1H, NH), 10.67 (s, 1H, NH), 7.72 (s, 1H, Ar-H), 7.59 (d, 1H, *J*= 8.4 Hz, Ar-H), 7.55 (d, 1H, *J* = 8.4 Hz, Ar-H), 2.26 (m, 6H, CH_2_), 2.08 (m, 3H, CH), 1.66 (m, 6H, CH_2_); ^13^C NMR (75 MHz DMSO-d_6_) δ (ppm)177.65 (C=S), 167.57 (C=O), 136.16, 133.81, 131.66, 131.07, 129.51, 127.75 (Ar-C), 54.90, 40.11, 36.20, 29.29 (adamantyl-C) Anal. calcd. for C_18_H_20_Cl_2_N_2_OS (383.33): C, 56.40; H, 5.26: Cl, 18.50: N, 7.31: O, 4.17: S, 8.36. Found: C, 56.41; H, 5.27; N, 7.32.

*N*-(Adamantan-1-ylcarbamothioyl)butyramide (**3c**):

White crystalline solid, m.p. = 136–137 °C, Yield = 90%, ^1^H NMR (DMSO-d_6_, 300 MHz,); δ (ppm): 12.67 (s, 1H, NH), 11.43 (s, 1H, NH), 2.26 (t, 2H, *J* = 2.3), 2.26 (s, 6H), 2.14 (s, 3H), 1.71 (s, 6H) 1.68 (sex, 2H, 2.2) 0.91 (t, 3H, CH_3_); ^13^C NMR (75 MHz DMSO-d_6_) δ (ppm) 181.52 (C=S), 168.09 (C=O), 54.71, 40.12, 37.2, 35.11, 29.12, 19,3 13,2 (*n*-propyl, adamantyl-C).) Anal. calcd. for C_15_H_24_N_2_OS (280.43): C, 64.25; H, 8.63: N, 9.99: O, 5.71: S, 11.43. Found: C, 64.26; H, 8.63; N, 10.01.

*N*-(Adamantan-1-ylcarbamothioyl)-4-methoxybenzamide (**3d**):

White crystalline solid, m.p. = 178–179 °C, Yield = 84%, R_f_ = 0.63 (n-Hexane: Ethyl acetate 4:1) ^1^H NMR (DMSO-d_6_, 300 MHz,); δ (ppm): 11.40 (s, 1H, NH), 10.20 (s, 1H, NH), 7.94 (d, 2H, *J* = 8.5 Hz Ar-H), 7,21 (d, 2H, *J* = 8.5 Hz, Ar-H), 3.84 (s, 3H, CH_3_) 2,25 (m, 6H, CH_2_), 2.07 (m, 3H, CH), 1.63 (m, 6H, CH_2_); ^13^C NMR (75 MHz DMSO-d_6_) δ (ppm) 177.35 (C=S), 167.63 (C=O), 135.17, 133.67, 131.26, 129.24, 127.12, 126.90 (Ar-C), 55.8 (CH_3_) 54.74, 40.15, 36.24, 29.25 (adamantyl-C). Anal. calcd. for C_19_H_24_N_2_O_2_S (344.47): C, 66.25; H, 7.02: N, 8.13: O, 9.29: S, 9.31. Found: C, 66.26; H, 8.14; N, 8.14.

*N*-(Adamantan-1-ylcarbamothioyl)-4-chlorobenzamide (**3e**):

White crystalline solid, m.p. = 182–183 °C, Yield = 86%, R_f_ = 0.57 (n-Hexane: Ethyl acetate 4:1) ^1^H NMR (DMSO-d_6_, 300 MHz,); δ (ppm): 11.51 (s, 1H, NH), 10.43 (s, 1H, NH), 7.96 (d, 2H, *J* = 8.5 Hz Ar-H), 7.65 (d, 2H, *J* = 8.5 Hz, Ar-H), 2,23 (m, 6H, CH_2_), 2.12 (m, 3H, CH), 1.70 (m, 6H, CH_2_); ^13^C NMR (75 MHz DMSO-d_6_) δ (ppm) 177.53 (C=S), 167.44 (C=O), 137.34,131.38, 130.13, 128.98 (Ar-C), 54.7, 40.17, 36.25, 29.2 (adamantyl-C). Anal. calcd. for C_18_H_21_ClN_2_OS (348.89): C, 61.97; H, 6.07: Cl, 10.16: N, 8.03: S, 9.19. Found: C, 61.98; H, 6.08; N, 8.04.

*N*-(Adamantan-1-ylcarbamothioyl)pentanamide (**3f**):

White crystalline solid, m.p. = 141–142 °C, Yield = 85%, ^1^H NMR (DMSO-d_6_, 300 MHz,); δ (ppm): 12.41 (s, 1H, NH), 11.32 (s, 1H, NH), 2.34 (t, 2H, *J* = 2.1), 2.21 (s, 6H), 2.09 (s, 3H), 1.51 (pent, 2H, *J* = 2.1), (s, 6H), 1.32 (sex, 2H, 2.2), 0.93 (t, 3H, CH_3_); ^13^C NMR (75 MHz DMSO-d_6_) δ (ppm) 182.12 (C=S), 167.91 (C=O), 54.61, 40.19, 36.41, 35.13, 30.01, 28.13, 21.71, 13.34 (*n*-butyl, adamantyl-C). Anal. calcd. for C_16_H_26_N_2_OS (294.46): C, 65.26; H, 8.90: N, 9.51: S, 10.89. Found: C, 65.27; H, 8.91; N, 10.90.

*N*-(Adamantan-1-ylcarbamothioyl)-2-chlorobenzamide (**3g**):

White crystalline solid, m.p. = 185–187 °C, Yield = 83%, R_f_ = 0.67 (n-Hexane: Ethyl acetate 4:1) ^1^H NMR (DMSO-d_6_, 300 MHz,); δ (ppm): 11.45 (s, 1H, NH), 10.51 (s, 1H, NH), 7.67 (d, 1H, *J* = 8.3 Hz Ar-H), 7,64 (t, 1H, *J* = 8.3 Hz, Ar-H),7,51 (t, 1H, *J* = 8.4 Hz, Ar-H),7,43 (d, 1H, *J* = 8.4 Hz, Ar-H), 2,27 (m, 6H, CH_2_), 2.09 (m, 3H, CH), 1.66 (m, 6H, CH_2_); ^13^C NMR (75 MHz DMSO-d_6_) δ (ppm) 177.22 (C=S), 167.49 (C=O), 134.21, 131.34, 132.15, 129.12, 128.17, 127.30 (Ar-C), 54.78, 40.18, 36.25, 29.31 (adamantyl-C). Anal. calcd. for C_18_H_21_ClN_2_OS (348.89): C, 61.97; H, 6.07: Cl, 10.16: N, 8.03, S, 9.19. Found: C, 61.98; H, 6.08; N, 8.04.

*N*-(Adamantan-1-ylcarbamothioyl)heptanamide (**3h**):

White crystalline solid, m.p. = 149–151 °C, Yield = 88%, ^1^H NMR (DMSO-d_6_, 300 MHz,); δ (ppm): 12.51 (s, 1H, NH), 11.39 (s, 1H, NH), 2.51 (t, 2H, *J* = 2.1), 2.29 (s, 6H), 2.14 (s, 3H), 1.67 (s, 6H),1.69 (sex, 2H, 2.2), 1.54 (pent, 2H, *J* = 2.22), 1,32-1.29 (m, 6H) 0.89 (t, 3H, CH_3_); ^13^C NMR (75 MHz DMSO-d_6_) δ (ppm) 181.33 (C=S), 168.21 (C=O), 54.55, 40.27, 36.43, 36.21, 31.14, 30.01, 28.32, 25.89, 22.61,14.17 (*n*-hexyl, adamantyl-C). Anal. calcd. for C_18_H_30_N_2_OS (322.51): C, 67.04; H, 9.38: N, 8.69: S, 9.94. Found: C, 67.05; H, 9.39; N, 8.70.

*N*-(Adamantan-1-ylcarbamothioyl)-3,5-dinitrobenzamide (**3i**):

White crystalline solid, m.p. = 210–211 °C, Yield = 82%, R_f_ = 0.42 (n-Hexane: Ethyl acetate 4:1) ^1^H NMR (DMSO-d_6_, 300 MHz,); δ (ppm): 10.95 (s, 1H, NH), 9.86 (s, 1H, NH), 9.01 (s, 2H, Ar-H), 8.90 (s, 1H, Ar-H), 2,22 (m, 6H, CH_2_), 2.16 (m, 3H, CH), 1.73 (m, 6H, CH_2_); ^13^C NMR (75 MHz DMSO-d_6_) δ (ppm) 181.52 (C=S), 168.09 (C=O), 148.92, 136, 129.45, 121.53, (Ar-C), 54.91, 40.27, 36.12, 29.41 (adamantyl-C). Anal. calcd. for C_18_H_20_N_4_O_5_S (404.44): C, 53.46; H, 4.98: N, 13.85: O, 19.78: S, 7.93. Found: C, 53.47; H, 4.99; N, 13.86.

*N*-(Adamantan-1-ylcarbamothioyl)octanamide (**3j**):

White crystalline solid, m.p. = 158–159 °C, Yield = 86%, ^1^H NMR (DMSO-d_6_, 300 MHz,); δ (ppm): 12.56 (s, 1H, NH), 11.38 (s, 1H, NH), 2.43 (t, 2H, *J* = 2.3), 2.32 (s, 6H), 2.18 (s, 3H), 1.67 (s, 6H) 1.55 (pent, 2H, *J* = 2.1), 1.32-1.28 (m, 8H), 0.89 (t, 3H, CH_3_); ^13^C NMR (75 MHz DMSO-d_6_) δ (ppm) 181.37 (C=S), 168.17 (C=O), 54.66, 40.21, 36.41, 36.19, 31.89, 30.07, 28.57, 25.91, 22.73,14.13 (*n*-heptyl, adamantyl-C).). Anal. calcd. for C_19_H_32_N_2_OS (336.22): C, 67.81; H, 9.88: N, 8.32; S, 9.53. Found: C, 67.82; H, 9.59; N, 8.32.

### 3.3. In Vitro Urease Inhibitory Activity

The amount of NH_3_ produced with indophenol’s method reported in the literature [28] was used to estimate Jack bean urease activity. In a 96-well plate, reaction mixtures containing 20 µL of Jack bean urease at 5 U/mL and 20 L of compounds in 50 µL of potassium phosphate buffer (100 mM urea, 10 mM K_2_HPO_4_, 1 mM EDTA, and 10 mM LiCl, pH 8.2) were incubated for 30 min at 37 °C. Briefly, 50 μL each of phenol reagents (1%, *w/v* phenol and 0.005%, *w*/*v* sodium nitroprusside) and 50 μL of alkali reagent (0.5%, *w/v* NaOH and 0.1% NaOCl were added to each well. The absorbance at 625 nm was evaluated after 10 min by utilizing a microplate reader (OPTI_Max_, Tunable). Each of the reactions was accomplished in triplicate. The urease inhibition activities were calculated using the following formula:

(1) $$Urease\;inhibition\;activity\;\left(\%\right)=\left(OD_{control}-OD_{sample}\times 100\right)/OD_{control}$$

where *OD_control_* and *OD_sample_* represent the optical densities in the absence and presence of sample, respectively, by using thiourea as standard.

### 3.4. Kinetic Analysis

Based on IC_50_ values, compound **3j** was identified as the most powerful. Changing the concentration of urea at different concentrations of **3j** (0.00, 0.0042, 0.0085, and 0.017 M) was used to study kinetics. For urease kinetics, the urea concentration was quickly adjusted from 100, 50, 25, 12.5, 6.25, and 3.12 mM. The highest preliminary velocities were determined from initial linear portion of absorbance up to 10 min after the addition of enzyme at per minute’s interval. The inhibition type was determined by Lineweaver-Burk plot (1/V) versus 1/[S] mM^−1^. The EI dissociation constant *Ki* was ascertained by secondary plot of 1/V against inhibitor concentration. Urease activity was revealed by measuring the amount of ammonia released. The results were processed by using SoftMaxPro.

### 3.5. Free Radical Scavenging Assay

The 2,2-diphenyl-1-picrylhydrazyl (DPPH) test was used to assess radical scavenging activity. By using ascorbic acid as a reference inhibitor, 100 µL of DPPH (150 M), 20 µL of escalating concentrations of test compounds, and the volume adjusted to 200 µL in each well with methanol were incubated for 30 min at room temperature. At 517 nm, an OPTI Max Tunable microplate reader was used to perform assay measurements. The % inhibition caused by the investigated inhibitors was calculated after response rates were compared.

### 3.6. Computational Methodology

#### 3.6.1. Selection of Jack Bean Urease Structure from PDB

The Jack bean urease structure from Protein Data Bank (PDB) (www.rcsb.org, accessed on 5 June 2021) with PDBID 4H9M was minimized by using UCSF Chimera 1.10.1 tool. Additionally, the stereo-chemical properties of urease structure and Ramachandran plot were obtained by Molprobity server [29] and Protparam [30]. The protein architecture, statistical percentage values of receptor proteins helices, beta-sheets, coils, and turn were anticipated by using VADAR 1.8 [31].

#### 3.6.2. Grid Generation and Molecular Docking

The co-crystallized ligands from Protein Data Bank and literature data [32] are used to define the enzyme’s active site. The docking experiment was carried out by utilizing the Glide docking methodology [33] against all produced ligands and the target protein. Using the Glide experiment, the predicted binding energies (docking scores) and conformational locations of ligands inside the active region of the protein were also discovered. Glide/SP/XP and induced fit docking (IFD) techniques accomplished both partial and full flexibility around the active site residues throughout the docking simulations [34].

## 4. Conclusions

The possibility of employing amantadine-thiourea conjugates derivatives as potent inhibitors of Jack bean urease was investigated in this study. When compared to ordinary thiourea, the entire series showed much more activity. Alkyl compounds were more powerful than their aryl analogues in general. Compounds **3d** with a 4-chloro substituent on the phenyl ring were found to be the most successful in the aryl series, while **3j** with a 7-carbon alkyl chain was shown to be very effective in the alkyl series. The kinetic experiments revealed a non-competitive inhibitory mode. According to the results of molecular docking, compound **3j** forms a stable complex with the target protein with low variation. Compound **3j** forms two hydrogen bonds with amino acid residue VAL391 having a binding distance of 1.858Å and 2.240 Å. The interaction of **3j** with amino acid residue located outside the catalytic site showed its non-competitive mode of inhibition. It is possible that compound **3j** will be used as a starting point for subsequent structure-based designs of improved urease inhibitors. However, more research is needed in order to confirm the effectiveness and safety of this new urease inhibitor.

## Figures and Tables

**Table 1:** Urease inhibitory activity and free radical % scavenging of compounds (**3a**–**j**).

Compound	Urease Activity IC_50_ ± SEM (µM)	Free radical % Scavenging (100 µg/mL)
**3a**	0.01 (0.001)	4.18 (0.03)
**3b**	0.01 (0.001)	1.46 (0.02)
**3c**	0.03 (0.001)	2.51 (0.00)
**3d**	0.02 (0.001)	1.86 (0.01)
**3e**	0.03 (0.002)	13.36 (0.01)
**3f**	0.03 (0.001)	4.26 (0.00)
**3g**	0.0087 (0.001)	1.12 (0.03)
**3h**	0.01 (0.001)	15.36 (0.01)
**3i**	0.01 (0.001)	5.91 (0.05)
**3j**	0.0085 (0.001)	9.96 (0.06)
**Thiourea**	4.74 (0.05)	------------------
**Vitamin C**	------------------	95.15 (0.05)

SEM = Standard error of the mean; values are expressed in mean ± SEM.

**Table 2:** Kinetic parameters of the jack bean urease for urea activity in the presence of different concentrations.

Dose (µM)	V_max_ (ΔA/Min)	Km (mM)	Inhibition Type	K*i* (µM)
0.00	0.013526993	4	Non-Competitive	0.007
0.0042	0.001560909	4		
0.0085	0.001224084	4		
0.017	0.001207695	4		

**Table 3:** Physiochemical properties of urease by ProtParam.

Sr. No.	Parameters	Values
1	Molecular weight (MW)	90,747.7 Da
2	Theoretical pI	6.05
3	Extinction coefficient * (assuming all Cys residues are reduced)	53,290
4	Aliphatic index	90.48
5	Instability index	31.75
6	Gran average of hydropathicity (GRAVY)	−0.152

* Extinction coefficient units M^−1^cm^−1^ at 280 nm.

**Table 4:** Glide energy score of docked complexes.

Docking Complexes	Glide Score	Glide Model
**3a**	−4.570	−40.098
**3b**	−4.278	−42.139
**3c**	−3.820	−33.323
**3d**	−4.515	−43.748
**3e**	−4.538	−40.909
**3f**	−4.538	−37.182
**3g**	−4.335	−40.851
**3h**	−4.868	−43.500
**3i**	−5.122	−49.540
**3j**	−4.746	−41.113

## Data Availability

Not applicable.

## Supplementary Materials

The following are available online. Figure S1: Docking complex **3a**; Figure S2: Docking complex **3b**, Figure S3: Docking complex **3c**, Figure S4: Docking complex **3d**, Figure S5: Docking complex **3e**, Figure S6: Docking complex **3f**, Figure S7: Docking complex **3g**, Figure S8: Docking complex **3h**, Figure S9: Docking complex **3i**, Figure S10: ^1^H-NMR spectrum of **3a**, Figure S11: ^13^C-NMR spectrum of **3a**, Figure S12: ^1^H-NMR spectrum of **3b**, Figure S13: ^13^C-NMR spectrum of **3b**, Figure S14: ^1^H-NMR spectrum of **3c**, Figure S15: ^13^C-NMR spectrum of **3c**, Figure S16: ^1^H-NMR spectrum of **3e**, Figure S17: ^13^C-NMR spectrum of **3e**, Figure S18: ^1^H-NMR spectrum of **3h**, Figure S19: ^13^C-NMR spectrum of **3h**.
