# Supplementary material for: Exploring Amantadine Derivatives as Urease Inhibitors: Molecular Docking and Structure–Activity Relationship (SAR) Studies
Source: Molecules. 2021 Nov 25;26(23):7150. doi: 10.3390/molecules26237150 (PMC8658948; doi:10.3390/molecules26237150)
Supplement: Supplementary file 1 [file molecules-26-07150-s001.zip › molecules-1415374-supplementary.pdf]

Supplementary Materials

Exploring Amantadine Derivatives as Urease Inhibitors: Molecular Docking and Structure-Activity Relationship (SAR) Studies

Atteeque Ahmed [1], Aamer Saeed [1,*], Omar M. Ali [2], Zeinhom M. El-Bahy [3], Pervaiz Ali Channar [1], Asma Khurshid [1], Arfa Tehzeeb [4], Zaman Ashraf [5], Hussain Raza [6], Anwar Ul-Hamid [7] and Mubashir Hassan [8]

[1] Department of Chemistry, Quaid-I-Azam University, Islamabad 45320, Pakistan.
[2] Department of Chemistry, Turabah University College, Turabah Branch, Taif University, P.O. Box 11099, Taif 21944, Saudi Arabia
[3] Department of Chemistry, Faculty of Science, Al-Azhar University, Nasr City 11884, Cairo, Egypt
[4] Department of Pharmacy, Quaid-i-Azam University, Islamabad 45320, Pakistan.
[5] Department of Chemistry, Allama Iqbal Open University, Islamabad 44000, Pakistan.
[6] Department of Biological Sciences, College of Natural Sciences, Kongju National University, 56 Gongjudehak-Ro, Gongju, Chungnam 314-701, Republic of Korea
[7] Core Research Facilities, King Fahd University of Petroleum and Minerals, Dhahran 31261, Saudi Arabia.
[8] Institute of Molecular Biology and Biotechnology (IMBB), The University of Lahore, Lahore, 54000, Pakistan.

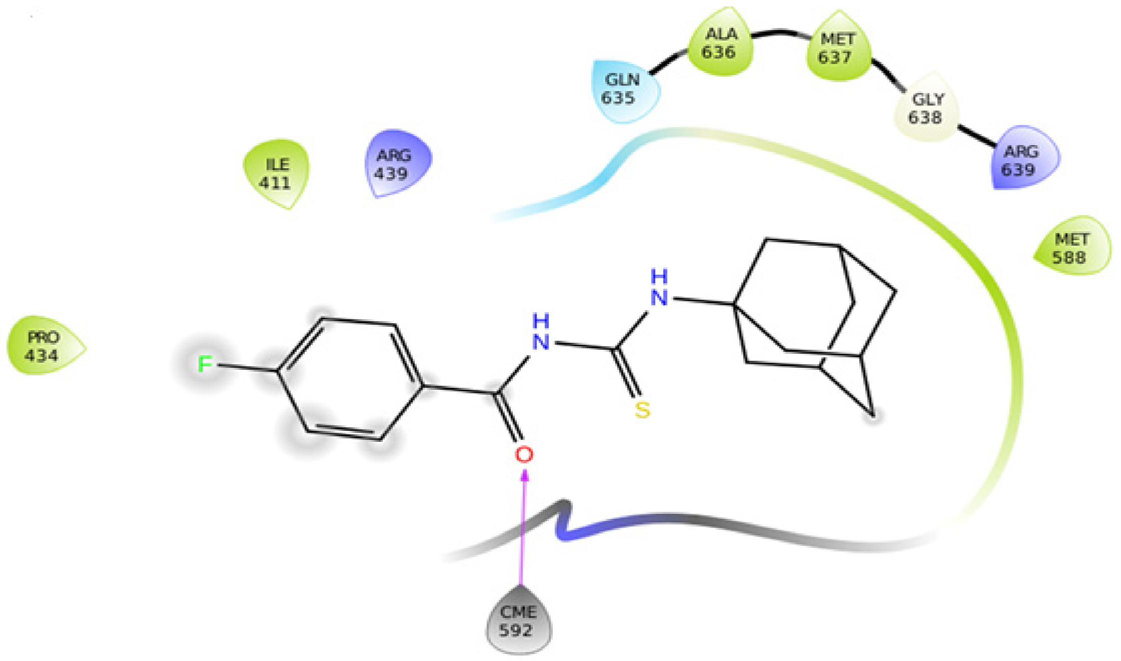

Figure S1. Docking complex **3a**

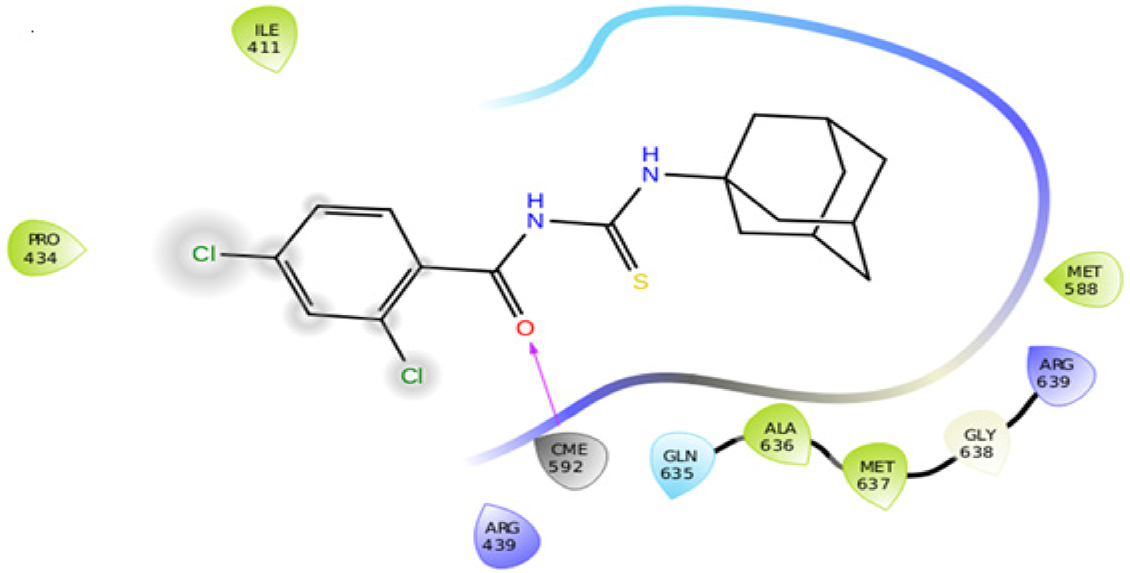

Figure S2. Docking complex **3b**

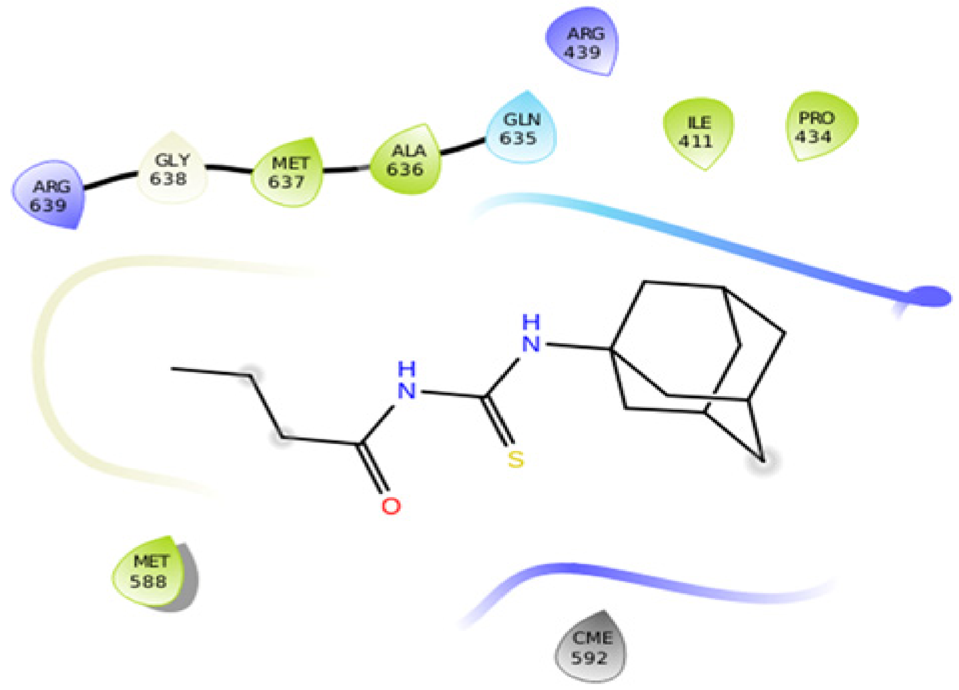

Figure S3. Docking complex **3c**

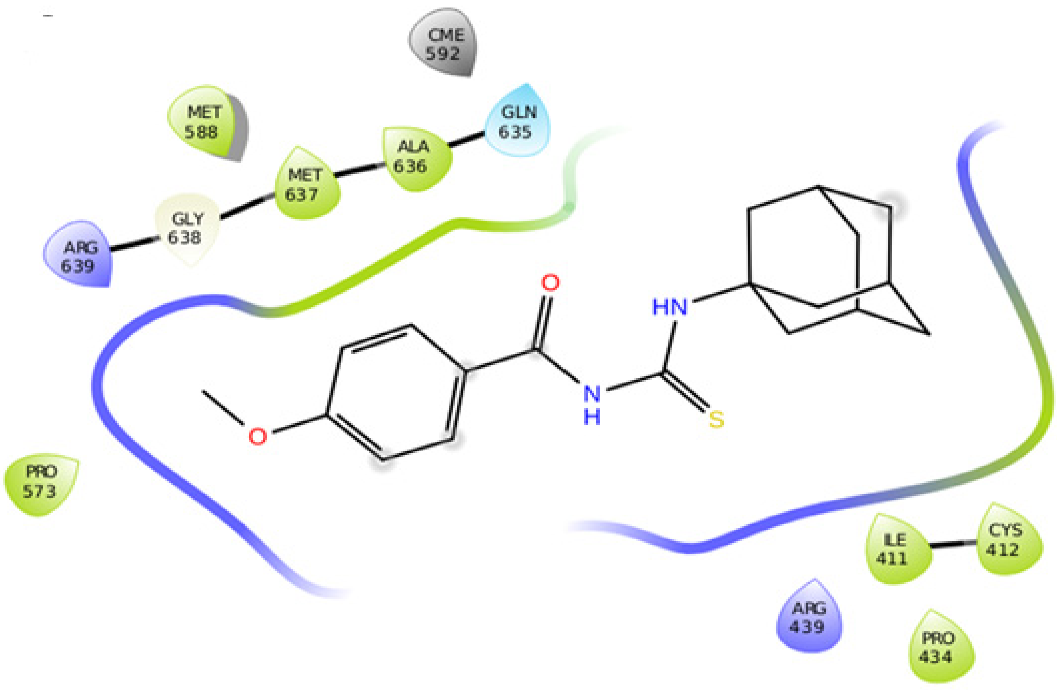

Figure S4. Docking complex **3d**

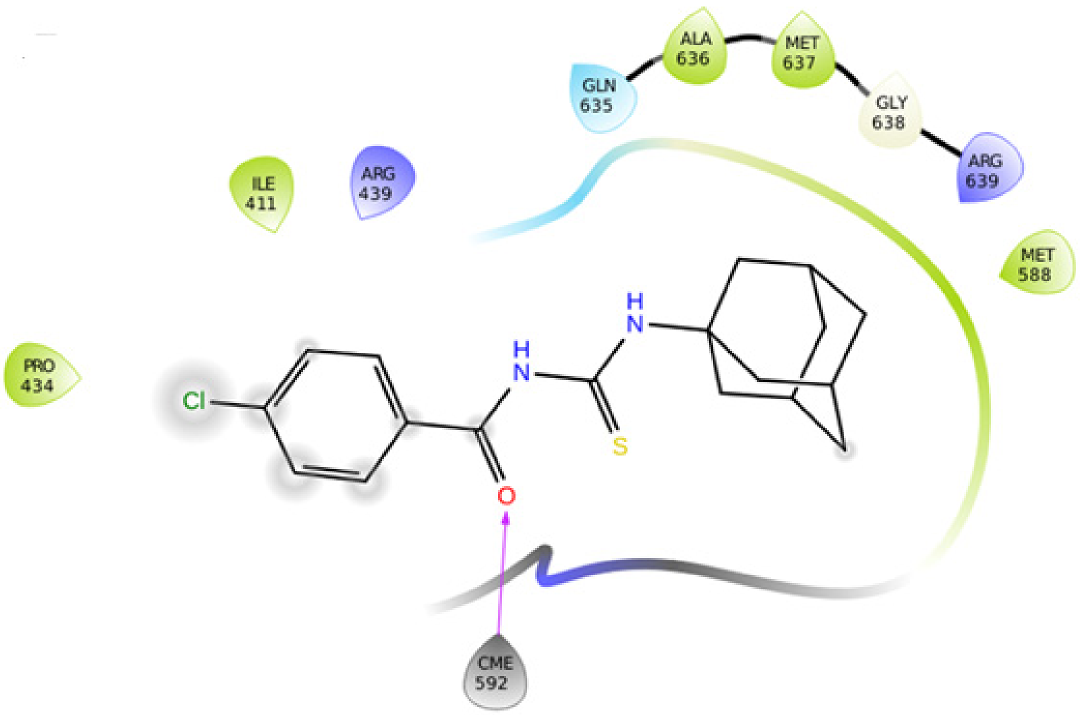

Figure S5. Docking complex **3e**

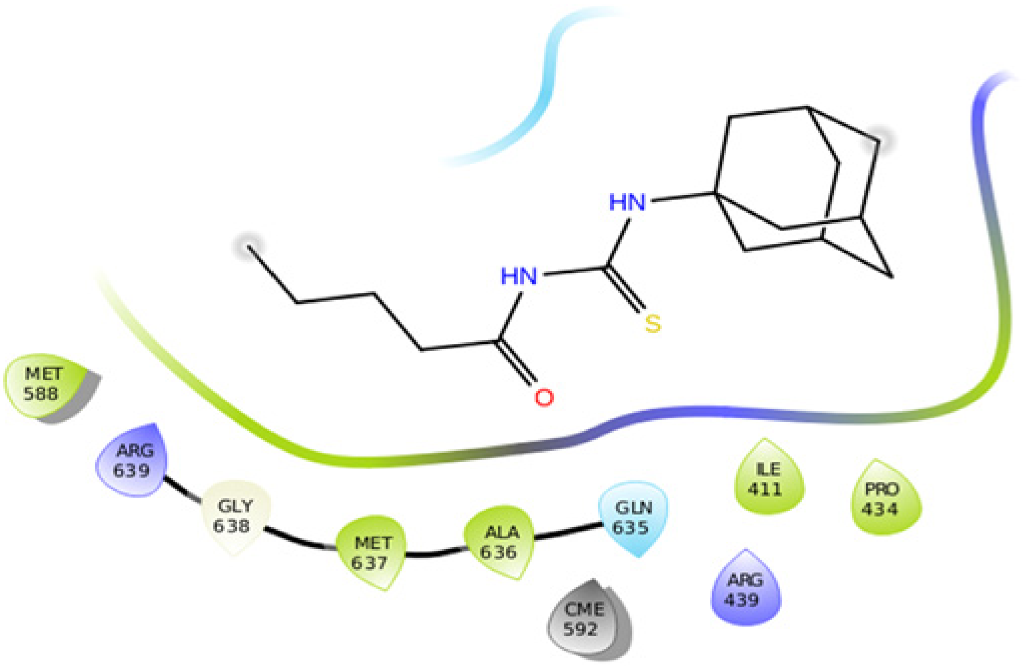

Figure S6. Docking complex **3f**

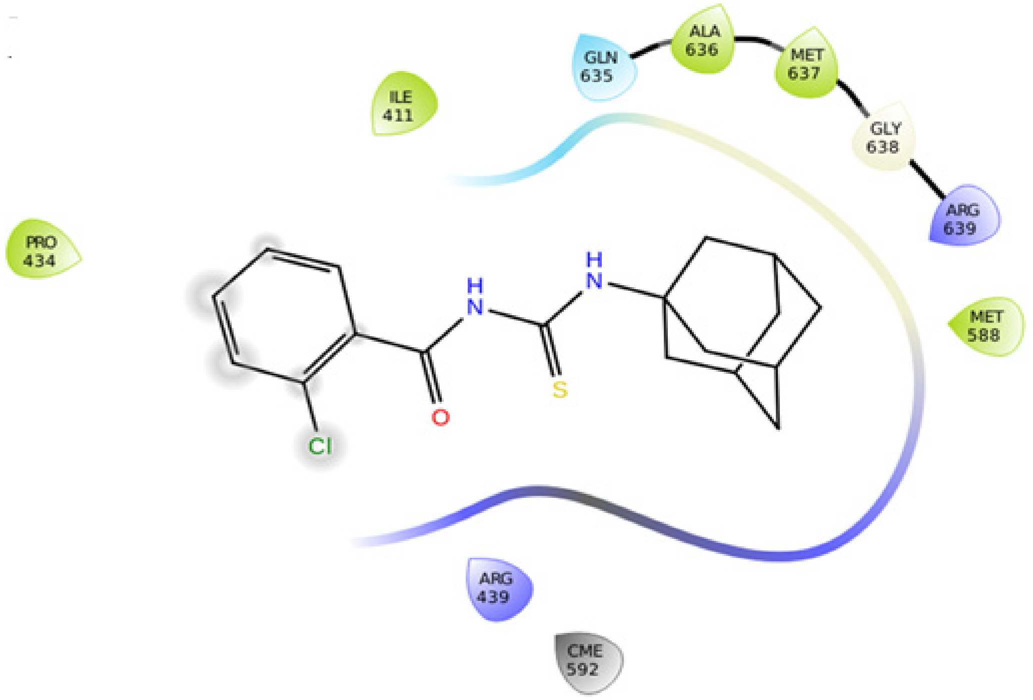

Figure S7. Docking complex **3g**

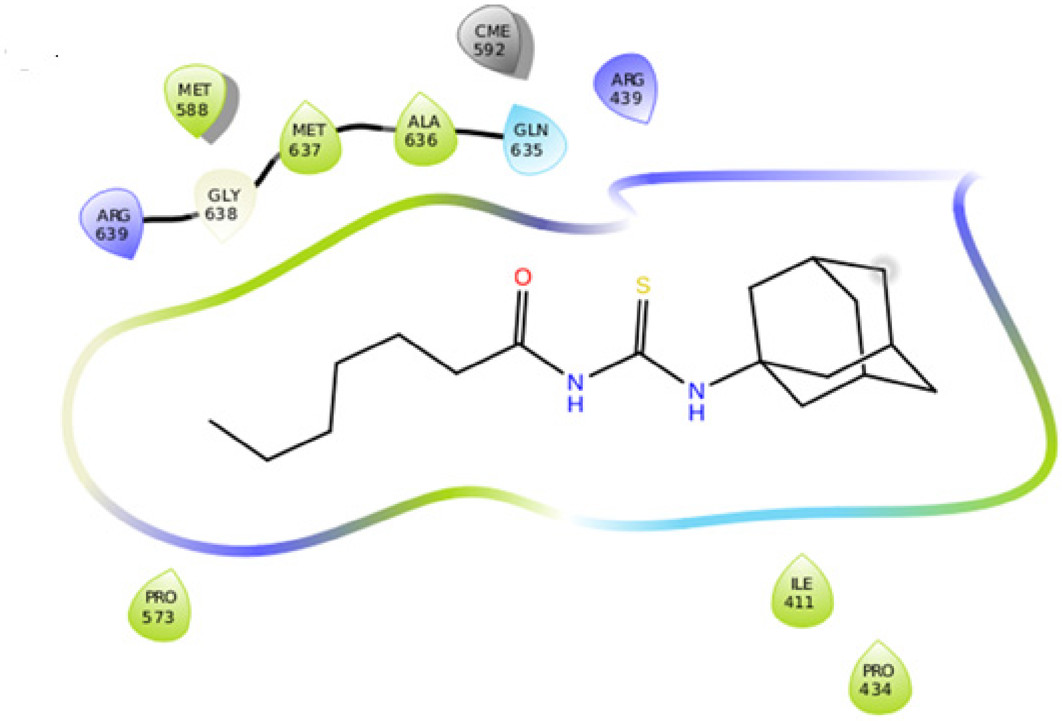

Figure S8. Docking complex **3h**

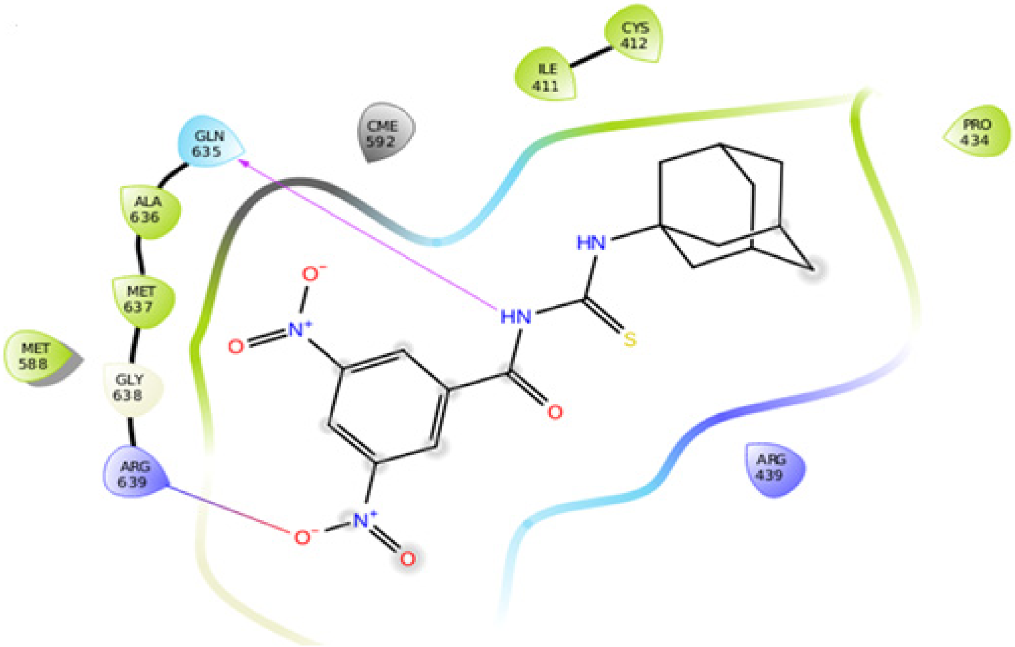

Figure S9. Docking complex **3i**

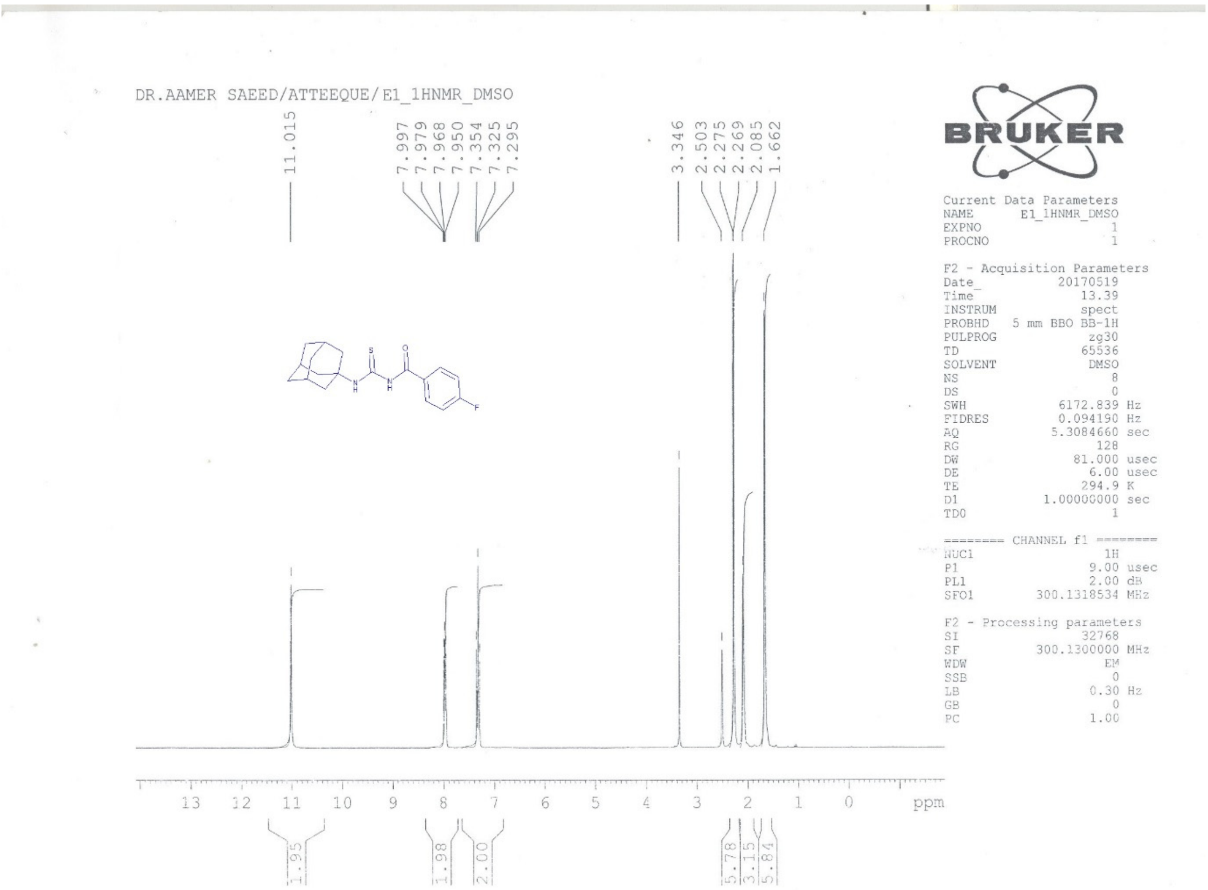

Figure S10. ^{1}H-NMR spectrum of **3a**

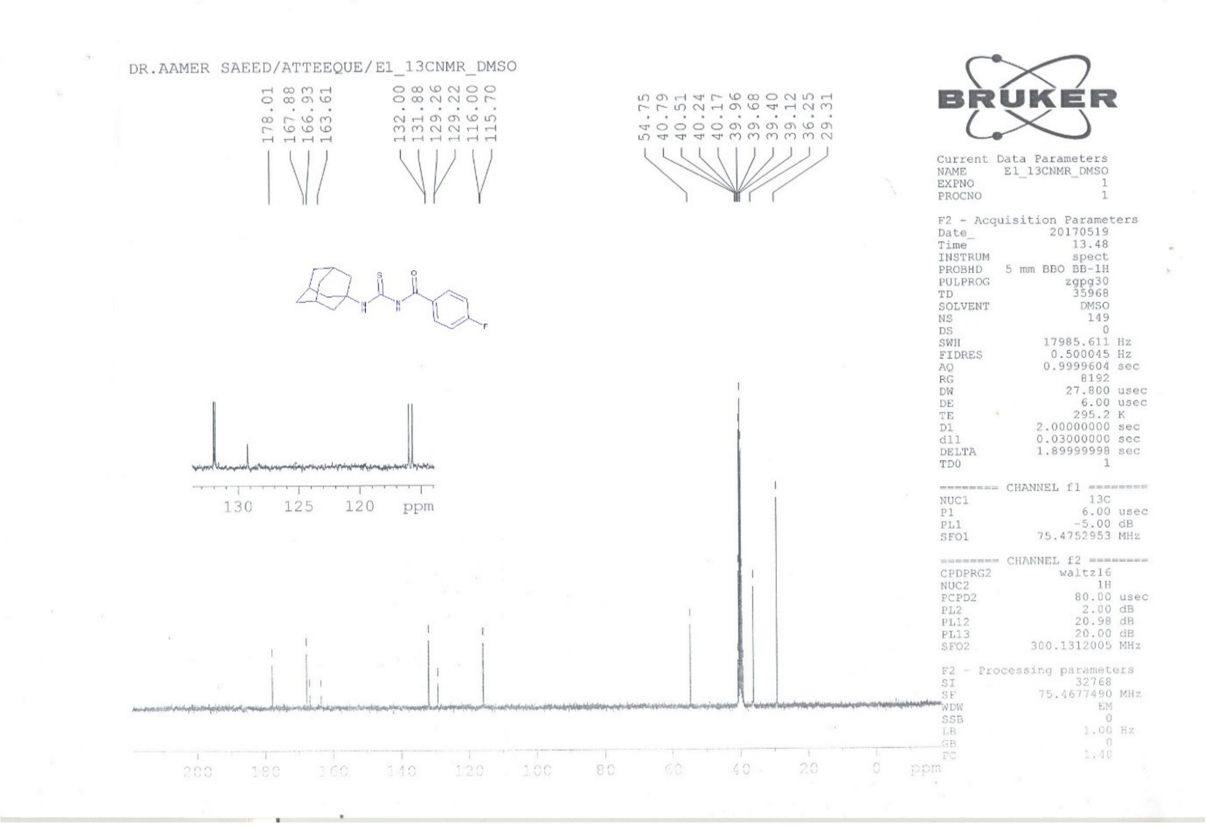

Figure S11. ^{13}C-NMR spectrum of **3a**

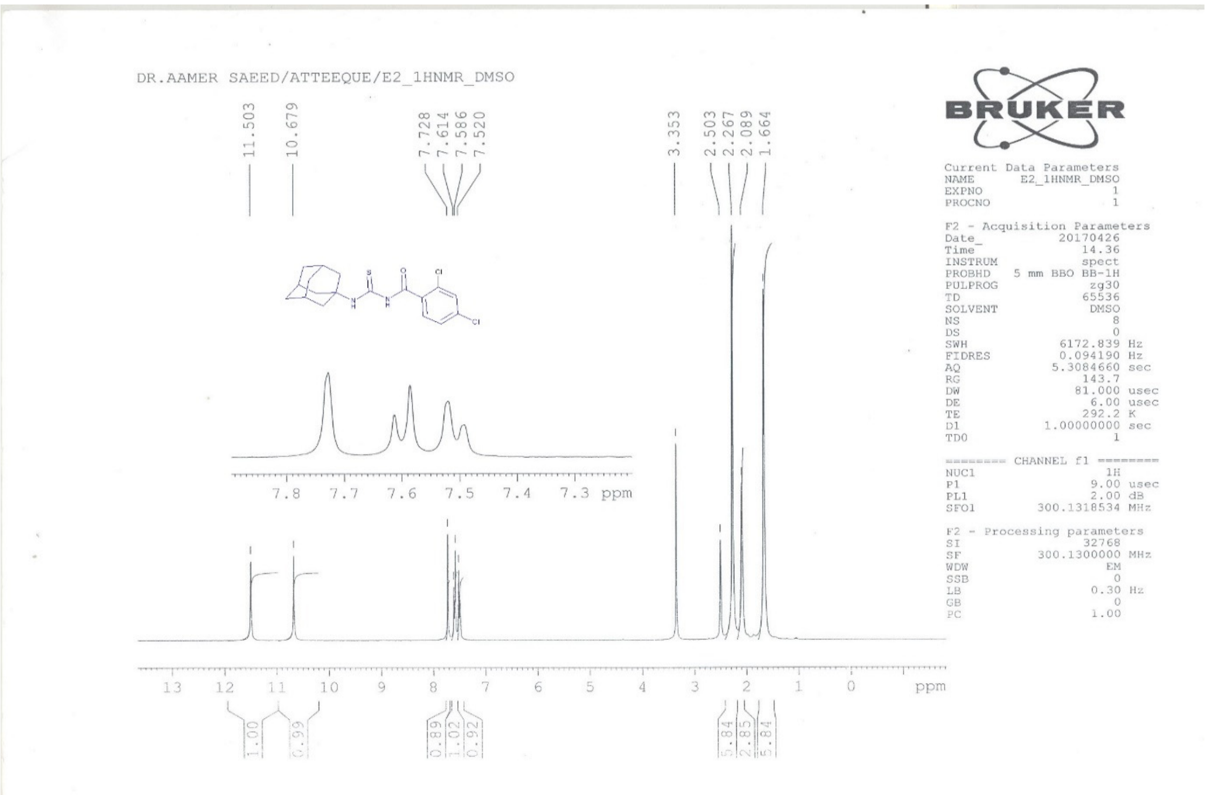

Figure S12. ^{1}H-NMR spectrum of **3b**

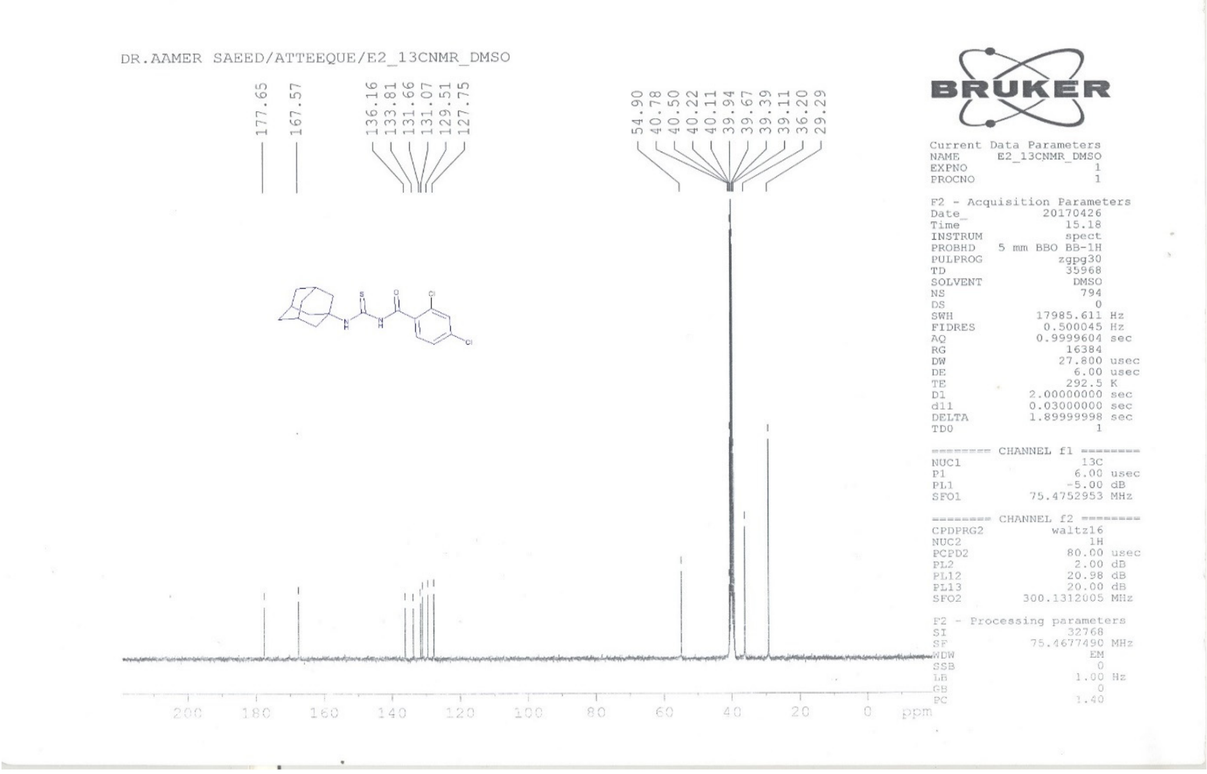

Figure S13. ^{13}C-NMR spectrum of **3b**

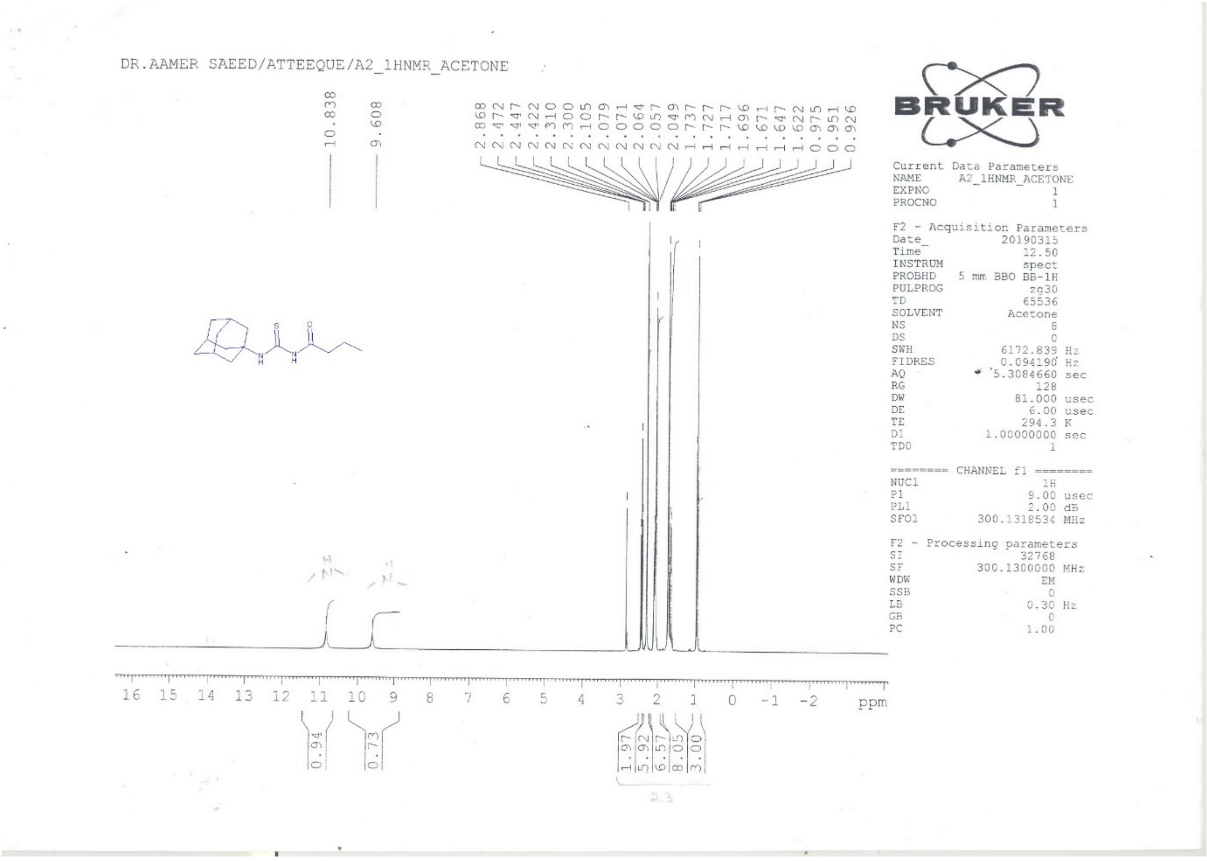

Figure S14. ^{1}H-NMR spectrum of **3c**

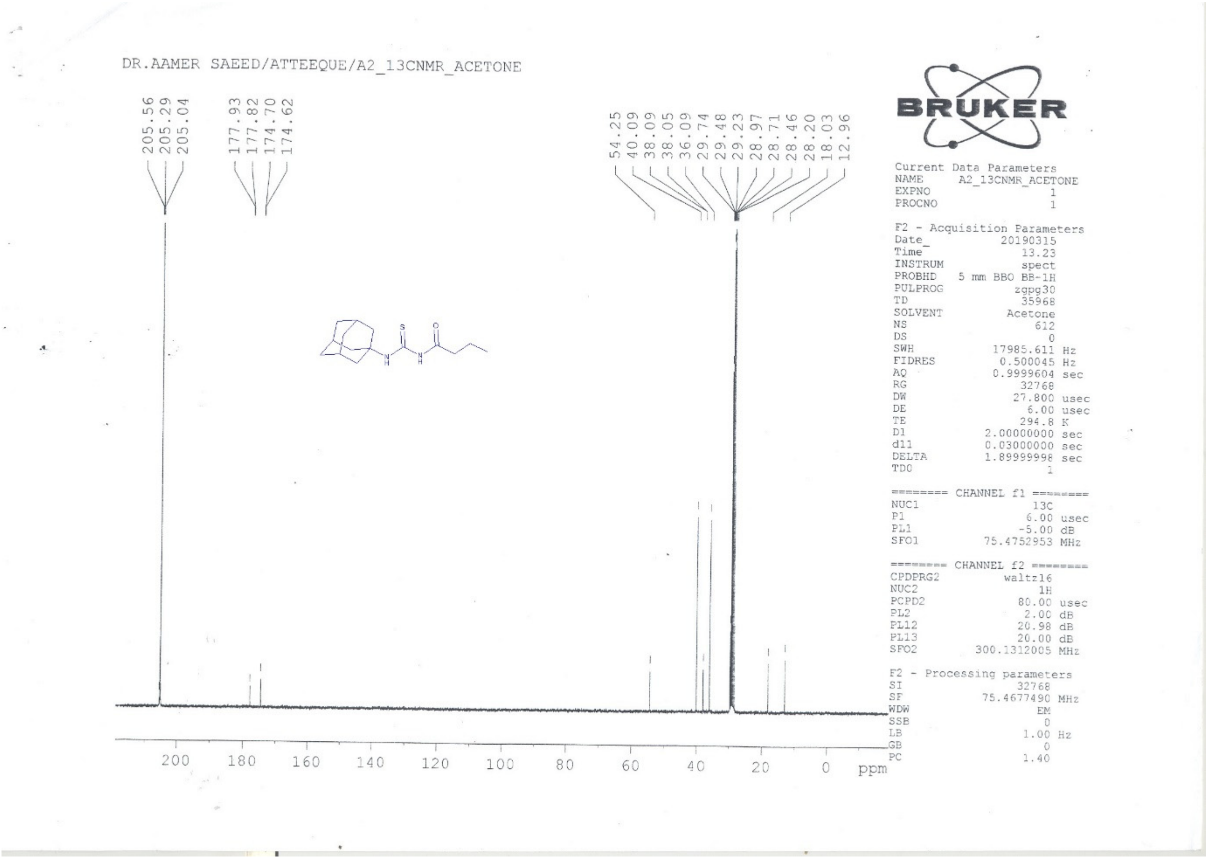

Figure S15. ^{13}C-NMR spectrum of **3c**

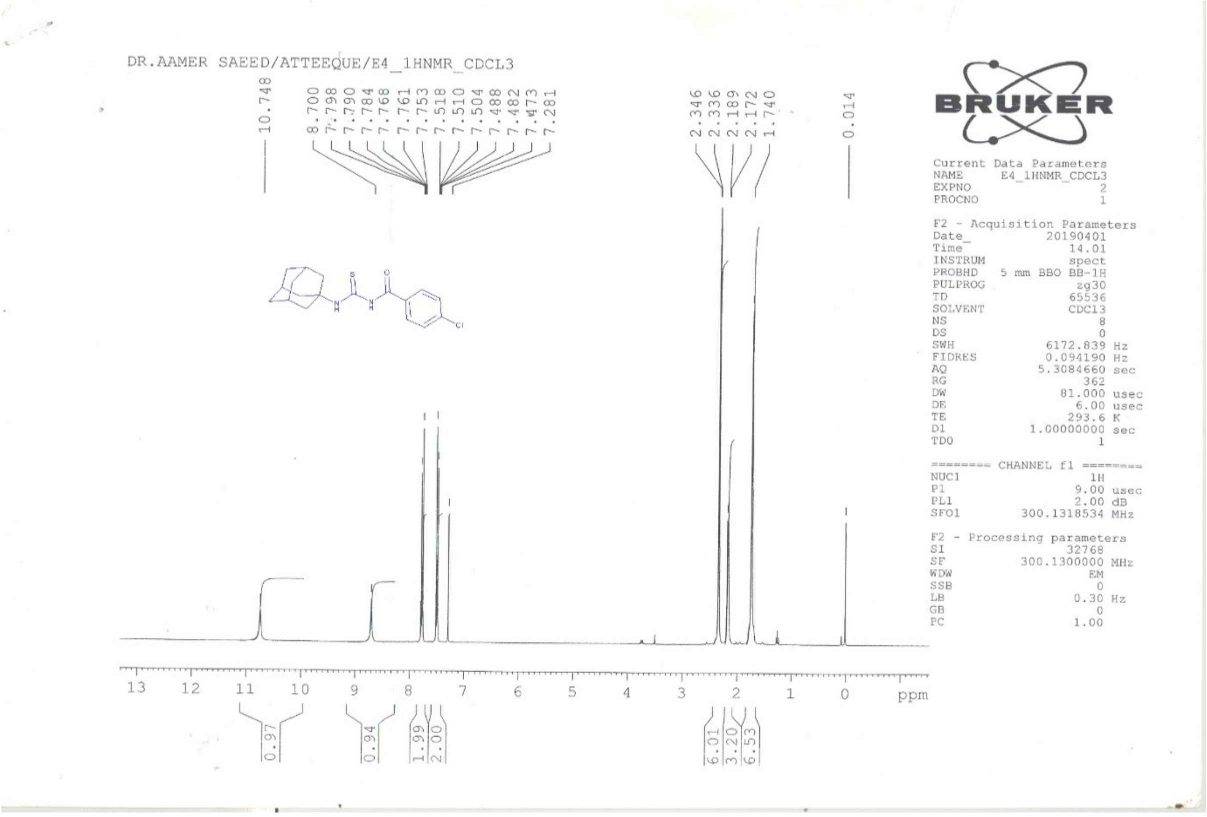

Figure S16. ^{1}H-NMR spectrum of **3e**

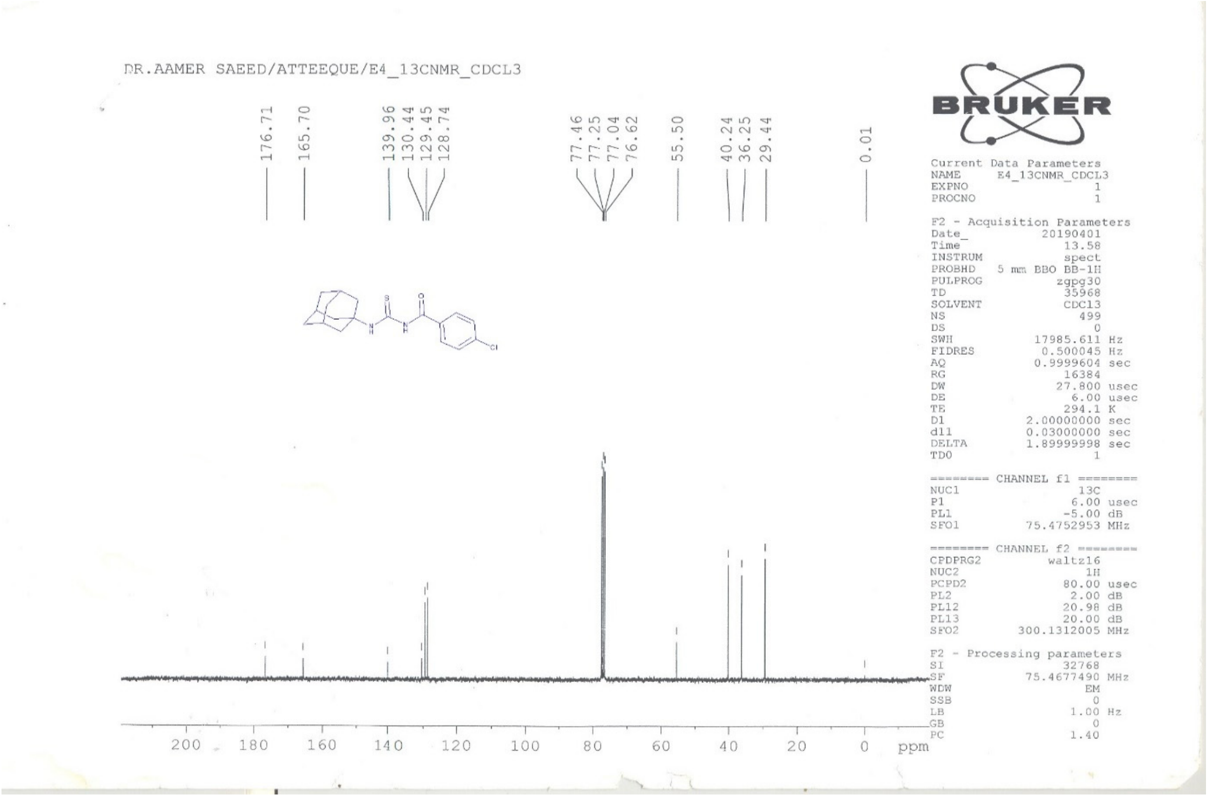

Figure S17. ^{13}C-NMR spectrum of **3e**

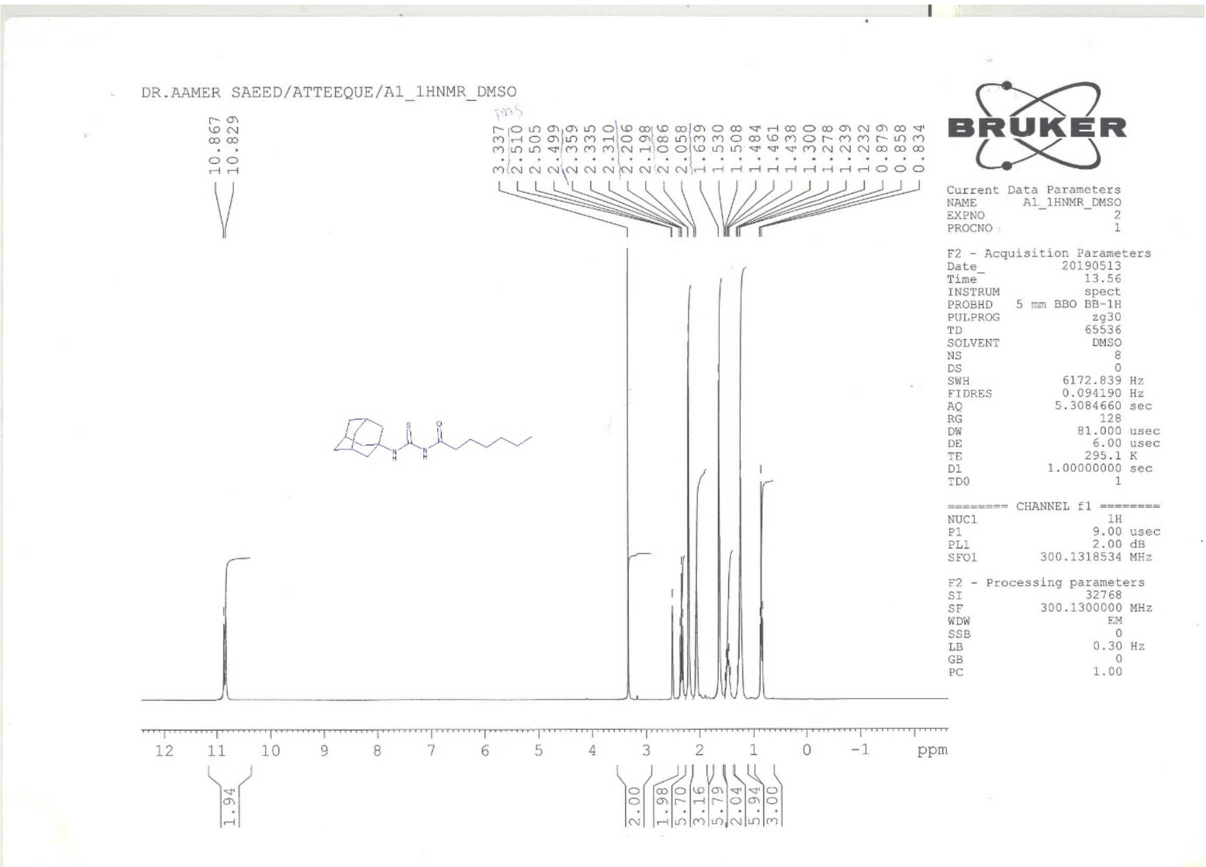

Figure S18. ^{1}H-NMR spectrum of **3h**

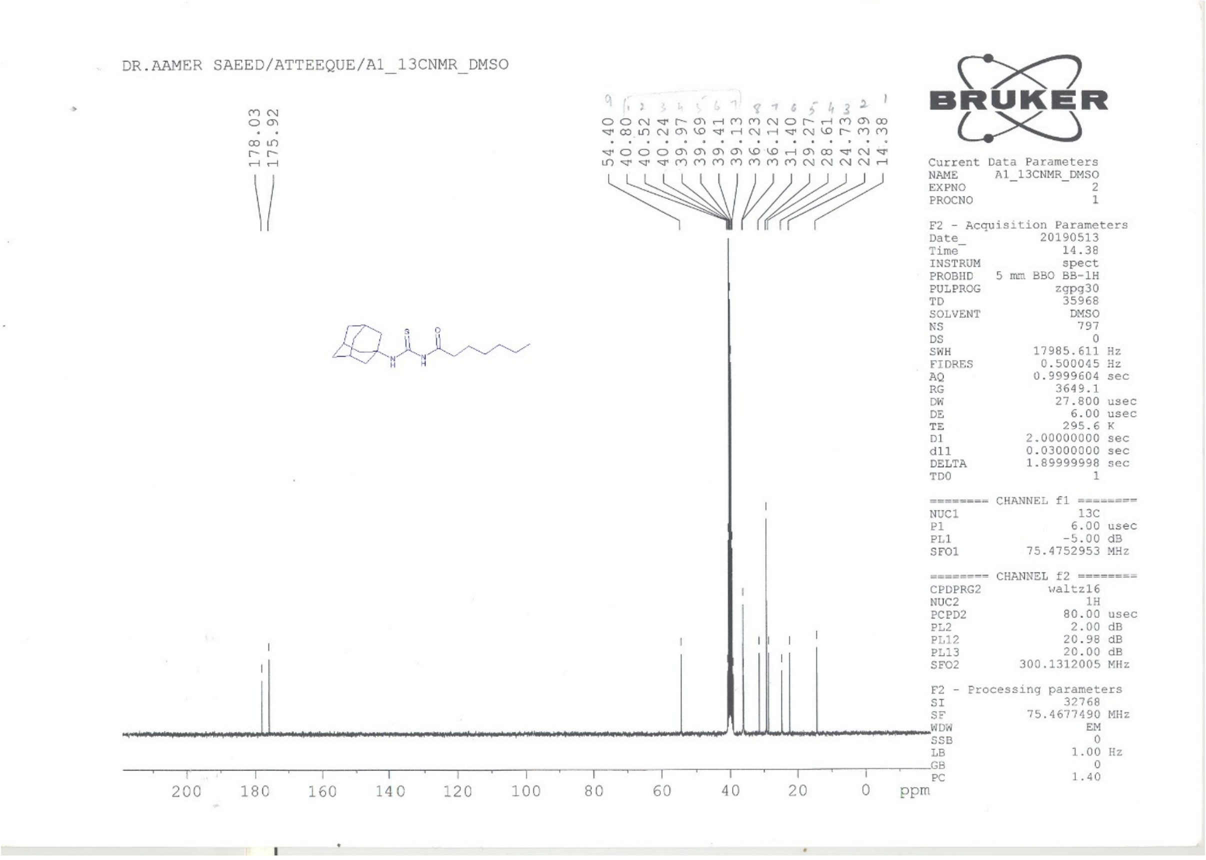

Figure S19. ^{13}C-NMR spectrum of **3h**
